# A Comparative Study of Automatic Localization Algorithms for Spherical Markers within 3D MRI Data

**DOI:** 10.3390/brainsci11070876

**Published:** 2021-06-30

**Authors:** Christian Fiedler, Paul-Philipp Jacobs, Marcel Müller, Silke Kolbig, Ronny Grunert, Jürgen Meixensberger, Dirk Winkler

**Affiliations:** 1 Department of Neurosurgery, University of Leipzig, 04103 Leipzig, SN, Germany; christian.fiedler@fh-zwickau.de (C.F.); ronny.grunert@medizin.uni-leipzig.de (R.G.); Juergen.Meixensberger@medizin.uni-leipzig.de (J.M.); Dirk.Winkler@medizin.uni-leipzig.de (D.W.); 2 Department of Physical Engineering/Computer Sciences, University of Applied Sciences, 08056 Zwickau, SN, Germany; silke.kolbig@fh-zwickau.de; 3 Fraunhofer Institute for Machine Tools and Forming Technology, 01187 Dresden, SN, Germany; marcel.mueller@iwu.fraunhofer.de

**Keywords:** magnetic resonance imaging (MRI), MRI marker, fiducial, automatic localization, sphere detection, segmentation, stereotaxy, bone anchor, neurosurgery

## Abstract

Localization of features and structures in images is an important task in medical image-processing. Characteristic structures and features are used in diagnostics and surgery planning for spatial adjustments of the volumetric data, including image registration or localization of bone-anchors and fiducials. Since this task is highly recurrent, a fast, reliable and automated approach without human interaction and parameter adjustment is of high interest. In this paper we propose and compare four image processing pipelines, including algorithms for automatic detection and localization of spherical features within 3D MRI data. We developed a convolution based method as well as algorithms based on connected-components labeling and analysis and the circular Hough-transform. A blob detection related approach, analyzing the Hessian determinant, was examined. Furthermore, we introduce a novel spherical MRI-marker design. In combination with the proposed algorithms and pipelines, this allows the detection and spatial localization, including the direction, of fiducials and bone-anchors.

## 1. Introduction

Processing volumetric medical image data plays a major role in diagnostics and surgery planning. Characteristic image features are extracted from the image data in order to register images stemming from different imaging modalities, or to determine a specific target point for a subsequent surgical intervention. In this context, the spherical feature shape is of particular importance. Due to its isotropic geometry, sphere-shaped features may serve as fiducial markers for positional alignment and image registration [1,2] independently of a specific imaging direction or relative positioning in the image.

Despite the fact that computer vision provides elementary methods for spherical and circular feature detection [3], the implementation in automatic image processing pipelines is rather difficult due to strongly use-case-specific parameter dependencies and required manual parameter adjustments. A comparitive study of different sphere detection approaches could provide essential information to develop automated workflows and prevent the necessity of a time consuming manual segmentation.

Image guided surgery is a particular application, where automated detection of spherical markers can be used for surgery planning [4]. Imaging techniques of various kinds, such as computer tomography (CT), T1- and T2-weighted magnetic resonance imaging (MRI) are used to obtain patient specific data for the surgery planning and guiding [5].

In order to benefit from the variety of information provided by multi modal imaging techniques, image registration is an essential step which aligns the coordinate systems of the pre-operative image space with the patient’s space, providing a mapping of points in the physical space with corresponding points in the radiographic image. Most registration algorithms based on anatomical landmarks are susceptible to image noise and differing anatomical information due to the particular imaging technique [6]. Spherical fiducial markers thereby provide the possibility of a robust and automated point-based registration.

In stereotactical surgery, bone anchors are applied near the location of surgery in order to mount stereotactical frames for navigation. Thus, besides image registration, the position and direction of bone anchors is of high interest during the operation planing process and may be determined by a suitable marker design.

Several approaches to point-based registration on extrinsic points already exist. Wang et al. used an intensity-based method to find centroids of tube-shaped fiducial markers attached to the patients head [7]. Among other approaches based on e.g., marker shape [8] or curvature [9], this technique lacks reliable directional information and therefore does not allow any sufficient assertion concerning the alignment of the applied bone anchors. Besides those CT- and MRI-based techniques for automatic marker localization, there are other methods requiring additional equipment including ultrasound [10], articulated arms [11], optical triangulation systems [12] or magnetic field digitizers [13].

In this paper, we propose four different approaches for automated localization of spherical features in 3D MRI data and compare them in terms of accuracy and robustness. The first algorithm is based on the circular Hough transform whereas the second is based on convolutional filtering. With the objective of an easy to use, parameter-free approach, we developed two more algorithms based on connected-components labeling and analysis, as well as on blob detection related methods and furthermore improved the first two algorithms accordingly. Moreover we present a novel fiducial marker design based on spherical landmarks, which allows for the determination of the positional and directional alignment of bone anchors in MRI data without any further X-ray examination. The proposed marker design additionally enables point based registration of multi modal MRI data.

## 2. Material and Methods

In the following section we introduce the novel marker model and design, the basic image processing pipeline, followed by a detailed description of the implemented algorithms.

### 2.1. Marker Model and Design

Two common concepts of fiducial marker attachment on the patients head exist. The first technique requires the pre-procedural placement of cranial bone anchors upon which the marker is screwed and is therefore considered invasive, while in the latter case the marker itself is attached non-invasively to the patient’s head using stickers. Invasive markers are used if bone anchors are needed for the further surgical procedure, such as navigation via stereotactical frames. In those cases, the location of the marker and the directional alignment of the underlying screw is interesting for further attachment of surgical frames and instruments.

**Figure 1:**
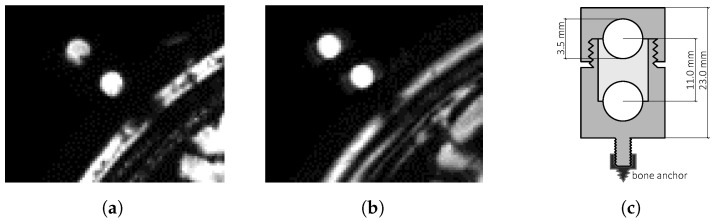
Marker depicted in (**a**) T1-weighted and (**b**) T2-weighted MRI and (**c**) as schematic drawing.

We developed a novel marker design (Figure 1c) consisting of a cylinder-shaped, rapid prototyped housing with two spherical cavities (r=3.5mm), aligned with the rotational axis of the cylinder and subsequently filled with MRI active substances. For the experiments in this paper, we used cholecalciferol, a vitamin D3 preparation in the form of a liquid filled oral capsule as MR contrast agent. The housing was manufactured using the biocompatible material polyether ether ketone (PEEK). The marker was designed to fit a commercial titanium bone-anchor with an internal metric M3 standard thread, manufactured by FHC, Inc., Bowdoin, ME 04287, USA. Figure 1a,b depict a typical T1- and T2-weighted MRI in which the spheres can be identified as circular structures with high intensity. By finding the sphere’s centroids and applying inherent geometrical constraints, it is possible to find corresponding pairs and subsequently determine the directional alignment by calculating the vector between the three-dimensional coordinates of the centroids. This procedure will be referred to as applying the marker model. A marker pair is built by picking a sphere from the set of unpaired marker sphere candidates and searching for a second sphere in the remaining set, fitting the distance threshold. The successfully paired marker spheres are removed from the set of marker sphere candidates. Accordingly, the marker model filters the sphere candidates by discarding potential false positive detected solitary spheres that do not have a corresponding partner within the given distance threshold of (11.0±1.0)mm.

### 2.2. Image Acquisition

We tested the proposed methods on data acquired on behalf of a *Philips Medical Systems Ingenia* MRI scanner at 3T. Five markers where attached upon previously placed cranial bone anchors on a human cadaver head. Anatomical fixation and conservation of the body donor was accomplished with ethanol-glycerin. Two different MRI imaging modalities, namely the T1 and T2 sequence, were conducted in order to show the capability of our marker detection methods. All MRIs were obtained in sagital orientation with isometric voxel sizes. In order to evaluate the influence of the spatial image resolution to the performance of the proposed algorithms, the voxel size was varied between (0.6mm×0.6mm×0.6mm) and (1.6mm×1.6mm×1.6mm) in 0.2mm steps. Consequently, the test data set consists of six MRI volumes per modality with a total number of 60 possibly detectable markers or 120 spheres, respectively. The acquired MRI data contains the full human cadaver head including brain, bone and skin tissue. The acquisition parameters were taken from the standard head MRI acquisition protocol for patients of the University of Leipzig Medical Center.

### 2.3. Image Processing Pipeline

**Figure 2:**
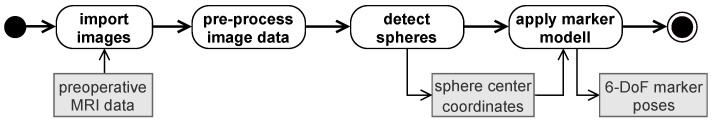
The general image processing pipeline.

In order to give an overview of the general workflow, Figure 2 shows the basic steps of the image-processing pipeline from gathering the pre-operative medical images to the final localization and classification of the detected markers.

The entire process can be separated into three basic steps. First, the previously acquired MRI and metadata has to be loaded and might undergo some image pre-processing steps, like re-sampling and noise removal.

The second step describes the actual localization of the spherical markers centroids. It varies, depending on the method chosen out of the four approaches mentioned earlier. Each approach is comprised of several computational steps and is described in its own section below.

After localizing the spheres, the third and final step is to match sphere pairs and refine the results by distinguishing false positives from actual sphere candidates when applying the marker model. The final output of the pipeline is a list of detected MRI markers and their pose, including position and spatial orientation.

In order to analyze the method’s robustness with respect to different MRI-measurement settings, each is associated with different levels of noise and image distortion, no data set specific manual parameter optimization was conducted. The sphere detection approaches are designed in an adaptive manner, automatically adjusting their parameters, such as the image resolution dependent kernel size.

### 2.4. Circular Hough Transform Approach

The Hough transform and its variations are known for being robust methods for line and curve detection in images. The circular Hough transformation (CHT) is a modified version that aims on finding circular shapes [14]. Basically, points in the image space are transformed into a set of votes for points within a discrete parameter space. The parameter space is defined according to the shape of interest. An accumulator array is used to gather the number of votes for each point in the parameter space. A high vote count for an accumulator element indicates the presence of a shape instance, which is characterized by the associated parameter combination.

In CHT, the shape of interest is a circle. It can be described by the equation ${\left(x-x_{c}\right)}^{2}+{\left(y-y_{c}\right)}^{2}=r^{2}$, where $x_{c}$ and $y_{c}$ denotes the center and *r* the radius of the circle. Hence the parameter space belongs to $R^{3}$. For each edge point $I_{i,j}$ in image space, votes are populated by additively drawing a circle ${\left(x-i\right)}^{2}+{\left(y-j\right)}^{2}=r^{2}$, centered at the edge point, on the accumulator’s x-y plane. This is done for every radius in parameter space, such that the radii increase along the accumulator’s z-axis. For a specific radius, high vote counts in the x-y plane correspond to the center parameters of detected circles.

Although variations of the Hough transformation generalized to 3D are available, they typically use a computationally costly higher-dimensional parameter space, requiring normal information to be available [15] or distinctive structured objects to leverage local image features [16].

**Figure 3:**
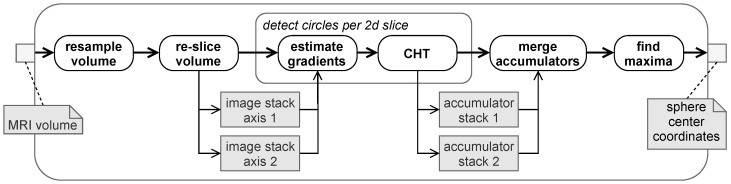
Pipeline for sphere detection based on circular Hough transform (CHT).

In this paper, we have chosen a bidirectional 2D-CHT approach (Figure 3) for sphere detection. Since MRI volumes often show an anisotropic voxel-size, the markers would appear as elliptical discs rather than circular ones. In case of anisotropic voxels, a pre-processing step is conducted to re-sample the MRI volume to an isotropic voxel size.

Subsequently, two stacks of slice images are extracted for sphere detection. Either stack contains images perpendicular to one of the orthogonal axes of the MRI volume. A first order derivative of Gaussian is used for edge detection within the slices. Since gradient directions are also estimated, accumulator drawing effort in the conducted CHT is reduced, as proposed in [17]. For both stacks, the CHT is performed independently and slice-wise in 2D.

The radius of our markers’ spheres is known a priori. Thus we limit the parameter space to a small set of radii, as suggested in [18] and collect the results in a 2D accumulator. Resulting per slice 2D accumulator images are reassembled into a 3D volume. Eventually, the two accumulator volumes are merged. Using multiplication as a blending method reinforces the intensity peaks in the accumulator map of spheres compared to other objects, e.g., a cylinder, that could also be depicted as a circle in a certain slice direction. Finally, we determine sphere centers by finding local maxima greater than a certain threshold in the accumulator and map their coordinates back to the physical coordinates of the original MRI volume image. This method will be further referred to as Hough method.

### 2.5. Convolution-Based Approach

In image processing, convolution filters are commonly used for tasks like smoothing, sharpening and extracting local features, such as lines and edges from an input image, where the applications differ in the filter kernel. For 3D images, the discrete convolution of an image *I* at position i,j,k by a kernel *F* of size (2p+1)×(2q+1)×(2r+1) is given by

(1) $$I^{*}\left(i,j,k\right)=\sum_{u=-p}^{p}\sum_{v=-q}^{q}\sum_{w=-r}^{r}F\left(u,v,w\right)\cdot I\left(i-u,j-v,k-w\right)\;,$$

and p,q,r∈N. Areas in the convolved image, where the computation involves values from outside of the input image’s boundaries are skipped and cropped respectively.

In MRI, the marker spheres are depicted as high intensity regions surrounded by a low intensity environment, due to the marker housing. In this approach, we treat the spheres as local image features and adapt a 3D convolution kernel for sphere detection that produces peaks at sphere centers in the convolved image. Hence, sphere positions can be determined by finding local maxima within the convolved image.

**Figure 4:**
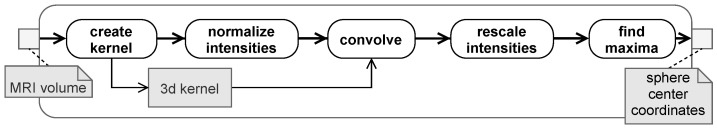
Pipeline for convolution-based sphere detection.

The first step in the filter pipeline (Figure 4) is creating a kernel. In accordance to the physical spheres and their extent in terms of voxel coordinates, kernel size and shape is chosen such that the sphere fits completely into the kernel. The kernel shape may vary from cubic, for isotropic, to cuboidal for anisotropic voxels. A typical kernel size is 7×7×7 for isotropic voxels at 1mm voxel size. Kernel elements are set to positive values for elements lying within an imaginary sphere placed at the kernel center (inside elements) and to negative values for outside elements. Intensity values of the input image are normalized to μ=0,σ=1, to increase robustness to changes in the intensity range, where μ is the mean of the intensity distribution and σ its standard deviation. Specifically, with the input image being normalized, we set the inside elements of the kernel to $k_{\mathrm{in}}=1.0$. Depending on the voxel spacing $s_{v}$, the value of the outside elements $k_{\mathrm{out}}\;\in \;\left(-1.5,-0.5\right)$ is chosen according to Equation (2) with steepnest parameter c=3.

(2) $$k_{\mathrm{out}}=-\left(0.5+\frac{1}{1+e^{-c(1-s_{v})}}\right)$$

Subsequently, the convolution is performed. Since the kernel is symmetrical, the operation could also be referred to as cross-correlation. As the convolution operation generates results greater than the range of input values, the result is re-scaled to an appropriate interval. Eventually, sphere centers are determined by finding local maxima exceeding a certain threshold. This method will be referred to as kernel method.

### 2.6. Connected Component Labeling and Analysis Approach

Connected component labeling (CCL) is a fundamental method for decomposing a binary image into a set of connected components, as introduced in [19]. The image is transformed into a symbolic image, designated as label map, such that a component is formed from each maximal connected subset of foreground pixels (or voxels in 3D) and a unique label (positive integer value) is assigned to it [20,21]. Accordingly, components are spatially disjoint to each other.

Once the connected components are labeled, feature measures can be applied. The results are utilized for decision making, i.e., in this context to decide whether a component corresponds to a MRI marker sphere or not. This is referred to as connected component analysis (CCA).

**Figure 5:**
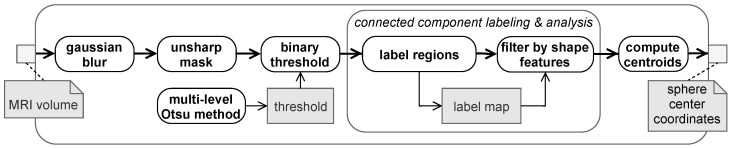
Pipeline for sphere detection based on connected component labeling and analysis.

The number and distribution of connected components, each considered as sphere candidate, mainly depends on the selected binary threshold and preliminary filtering steps. Ideally, a threshold is selected, such that a single connected component is corresponding to the physical spheres, each. Thereby, the total number of connected components, i.e., spheres and non-spheres, is supposed to be as low as possible. Thus, the sphere detection pipeline (Figure 5) involves some preprocessing steps.

A Gaussian filter is applied first for noise cancelation. Unblurred processing tends to produce several tiny components and clutter inside the head area. Due to the blurring step or blurry original images, nearby spheres easily merge together or with the head. This occurs if the contrast between spheres and their background (the housing) is to low, i.e., if they are not surrounded by voxels having a sufficiently low intensity. The unsharp mask filter in step two locally increases edge contrast and in particular decreases the intensity of the spheres environment. In the next step, the volume is segmented by binary thresholding, whereas the volume is split into foreground (i.e., head and marker spheres) and background voxels by a specified intensity threshold value. One may consider using the Otsu method [22] for automatic threshold selection. The binary threshold determined by the method tends to clutter the head area into several fragments, causing many sphere candidates to be created by subsequent CCL. While it still performs well in local regions around a marker, it might be ineligible for sphere detection in the entire volume. For the latter case instead, multi-level Otsu method [22] is utilized for threshold selection. The method segments a volume into an arbitrary number of classes (i.e., multiple thresholds) while maximizing the between-class variance of intensities, which is equal to k-means clustering performed on intensity histogram [23]. The pipeline involves a four-class Otsu method for automatic threshold selection, whereas the first class is considered as background and the other classes as foreground, accordingly. Since the pipeline solely relies on a binary segmentation, only the thresholding of the first class is used.

Next step comprises connected component labeling and analysis. In the labeling part, foreground voxels are grouped such that 6-adjacent voxels share the same group and each disjoint group is labeled with an unique identifier. Each of the components created is considered a sphere candidate, hence CCL acts as a hypotheses generator. After that, in CCA, candidates that do not resemble a marker sphere are discarded. Marker spheres are characterized by radius and a roundness measure. Roundness is defined as described in [24] by the ratio between the surface area of a hypothetical sphere, having the same volume as the sphere candidate, and the surface area measured on the candidate. Sphere positions are finally determined by computing the centroid of the remaining components. This method will be referred to as CCA method.

### 2.7. Blob Detection Related Approach

Blob detection is a method to find a compact region in a digital image which is lighter (or darker) than its background surrounded by a smoothly curved edge [25]. Since the spherical markers appear as circular structure with a homogeneous intensity surrounded by a dark area, blob detection seems to be an appropriate instrument to detect them. There are mainly three different methods, namely the Laplacian of Gaussian, the difference of Gaussian and the Determinant of Hessian (DoH) used for blob detection [26]. Because of its high precision and computational efficiency, the DoH outperforms the other methods [27,28] and thus was the method of choice in this paper and will be described below. In this paper, blobs are produced by the intensity contrast of the MRI marker spheres with a predefined uniform radius, *r*. Thus, only a single chracteristic scale index *t* must be seleceted in order to detect the blobs of interest. The scale index can be set into relation with the blob radius by $r=\sqrt{2t}$ [29]. For convenience, size in context of the proposed method means the distance between voxels i.e., units of voxel spacing.

Consider each slice in a MRI as a discrete function, I(x,y), representing an intensity value in every point or pixel in the image. The scale-space representation L(x,y;t) of *I* at the scale index *t* is derived from I(x,y) through a discrete Gaussian-smoothing operation by a convolution of I(x,y) with

(3) $$G\left(x,y;\sigma \right)=\frac{1}{2\pi \sigma^{2}}e^{-\frac{x^{2}+y^{2}}{2\sigma^{2}}}\;,$$

where $\sigma^{2}=t$ is the variance of the Gaussian distribution G(x,y;σ). Whereas, the derivatives magnitude decreases with increasing scale index *t*, they need to be normalized. This leads to the normalized Hessian feature detector operator, applied to the scale space representation L(x,y;t)

(4) $$\begin{array}{cc} detH_{norm}L & =det\left(\begin{array}{cc} t\partial_{xx} & t\partial_{xy}\\ t\partial_{yx} & t\partial_{yy} \end{array}\right)L\\ \; & =t^{2}\left(L_{xx}L_{yy}-L_{xy}^{2}\right). \end{array}$$

Originating from scale-space theory, the Hessian feature detector is build to find blobs of different scale, i.e., size, by finding the operators maximum response in scale-space. To convey the general idea of scale-space theory and scale-space selection, a detailed description is given in [30]. In order to find the marker centroid’s position, the task becomes detecting local extrema in the determinant response of the Hessian of the scale space representation, i.e., maxima in case of bright blobs, which would be at the blobs center. Therefore, local maxima in a marker-radius-sized neighbourhood of each voxel were detected and taken as possible marker position candidates.

Since the number of possible marker-candidates is in the order of magnitude of $n\;=\;10^{3}$, the need of a feature descriptor becomes obvious, in order to filter false positive results from actual markers.

**Figure 6:**
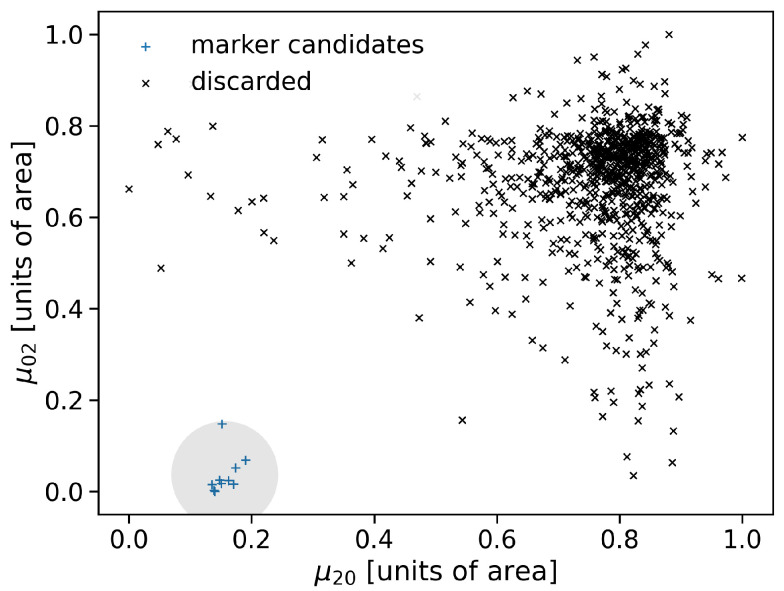
Normalized second order central image moments calculated for a marker-sized local area around marker candidates in the original MRI. Moments are calculated in three dimensions but plotted in two dimensions for visualization purposes. K-Means clustering was applied in order to distinguish false positive clusters from true markers. The cluster of marker candidates is highlighted by a circle around the clusters centroid.

To this end, an image-moment-based analysis of a local neighbourhood, with equal size as above, of the marker candidates in the original MRI was conducted. Since, the second order central image moment gives information about the intensity distribution around the center of the examined local area [31], the blob-like distributions of the actual markers should produce a characteristic value because of their equal size and shape. As can be seen in Figure 6, the actual markers form a cluster which is spatially separatable from falsely positive detected marker-candidates. K-means clustering was applied to classify the computed second order image moments according to their size. Thereby, the optimum number of clusters was found by the heuristic Knee-method [32]. The cluster whose mean value was closest to the characteristic size of the markers was chosen to contain the distributions originating from actual markers.

**Figure 7:**
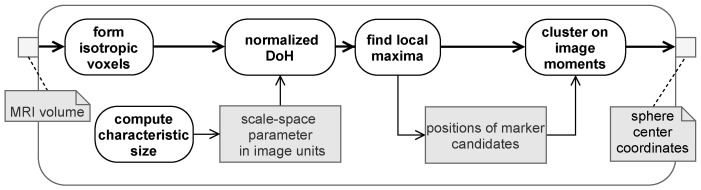
Pipeline for the blob-detection related approach.

Figure 7 shows a summary of the main components of the marker detection pipeline.

However, even after image-moment-based clustering, some false positive marker candidates remain in the selected cluster. Thus, the previously mentioned marker model was applied to successfully discard the residual falsely detected marker candidates. This proposed blob detection related approach will be referred to as blob method.

## 3. Results

**Figure 8:**
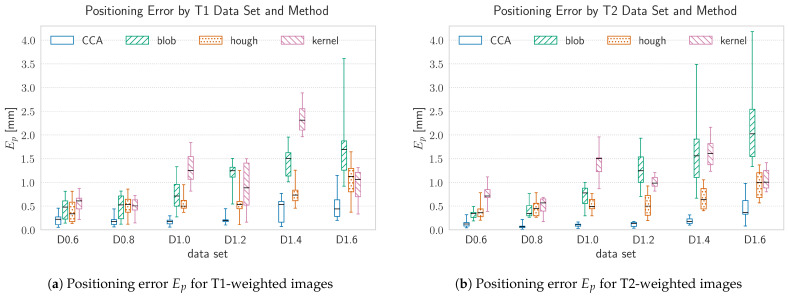
The positioning error, $E_{p}$, by data-set and method. $E_{p}$ was evaluated using the Euclidean distance between the positions of the detected markers to the ground-truth measurement. Each data-set was acquired at a different voxel size, ranging between s=[0.6,…,1.6]mm in Δs=2mm steps. The data-sets are ordered by increasing voxel size from left to right, where the data-set name annotates the voxel size in the following manner: Dx.x, with x.x denoting the voxel size in mm.

In order to evaluate the segmentation results of the proposed algorithms, manual segmentation was taken as ground truth. Manual segmentation was conducted by five scientific associates of the Department of Neurosurgery, University of Leipzig, trained in segmentation of medical images, using D2P (DICOM To Print) by 3D SYSTEMS Inc., Rock Hill, SC 29730, USA, a commercial image processing software. Coordinates of the segmented spherical volume centroids, averaged over the manual meassurements, were taken as marker sphere coordinates. The standard deviation of the manual measurements of the sphere centroid position was $\sigma_{gt}=0.37\;\mathrm{mm}$. The ground-truth measurement thereby served two purposes. By finding both, false positives in the set of marker candidates found by the algorithms and non detected markers, the ground truth is used to rate the classification capability of the proposed methods on the one hand. On the other hand, localization accuracy and precision of the algorithms were evaluated using the Euclidean distance between the positions of the detected markers to the ground truth measurement $E_{p}$, referred to as positioning error. Modality wise results are depicted in Figure 8a for T1-weighted and in Figure 8b for T2-weighted images, respectively.

**Figure 9:**
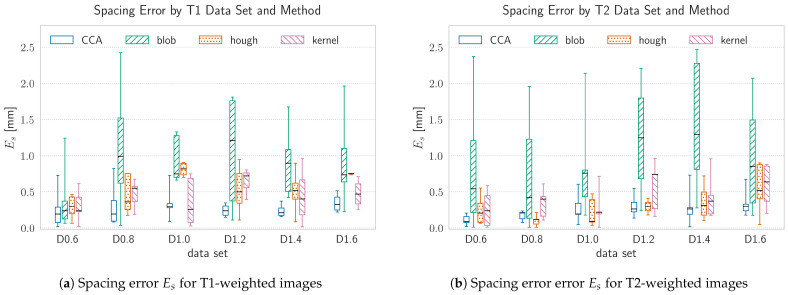
The spacing error, $E_{s}$, by data-set and method. $E_{s}$ reflects the deviation of the relative distance between two marker spheres of the same marker and the constructive-wise fixed distance value of 11mm. Since this error is evaluated upon marker model assumptions, it serves as a ground-truth bias independent measure for the spatial error. The mean spacing error of the manual ground-truth measurements across all data-sets was 0.22mm with a standard deviation of 0.17mm. Each data-set was acquired at a different voxel size, ranging between s=[0.6,…,1.6]mm in Δs=2mm steps. The data-sets are ordered by increasing voxel size from left to right, where the data-set name annotates the voxel size in the following manner: Dx.x, with x.x denoting the voxel size in mm.

Since the underlying manual segmentation result tends to be subjected to errors, due to the non automated manner of conduction, a further measure for localization accuracy and precision was taken into account. Therefore the deviation of the relative distance between two marker-spheres of the same marker and the constructive-wise fixed distance value of 11mm was evaluated, which will be referred to as spacing error $E_{s}$ and is depicted in Figure 9a for T1-weighted and in Figure 9b for T2-weighted images, respectively. Consequently, this measure can only be applied where both spheres have been recognized and will merely result in one value per two spheres.

Besides the geometrical measures, the $F_{1}$ score was calculated before and after application of the marker model to evaluate the classification accuracy of the proposed methods. Table 1 summarizes the number of true positive (*tp*), false positive (*fp*) and false negative (*fn*) results of the marker detection as well as the calculated $F_{1}$ score per data set and method.

(5) $$F_{1}=\frac{2}{{\mathrm{recall}}^{-1}+{\mathrm{precision}}^{-1}}=\frac{tp}{tp+\frac{1}{2}\left(fp+fn\right)}$$

The $F_{1}$ score, which is the harmonic mean of precision and recall, is given by Equation (5). A score of 1.0 means that there were no false positives or false negatives. It corresponds to the Dice coefficient [33] applied to Boolean data, using the definition of *tp*, *fp* and *fn*.

Regarding the computation time, CCA, Hough and kernel method range in the same order of magnitude, while blob detection is up to a factor of $10^{2}$ slower. For a typical head volume, e.g., the T1 data set with voxel size 1mm, blob detection requires up to 243.2s, whereas all other approaches finish between 1.4s and 3.3s of computation time, with CCA being the fastest method on standard consumer hardware.

## 4. Discussion

In the following section, we discuss the influence of the imaging modality, voxel size and the application of the marker model on the classification and localization capabilities of the proposed methods. Additionally, we discuss how the error in the position estimation affects the markers main axis estimation.

### 4.1. Influence of the Imaging Modality

The visibility of the marker spheres depends on the chosen modality, because T1- and T2-weighted MRIs serve different image contrasts concerning to different tissue types. Since the spheres consist of a fluid bulk, surrounded by a non-fluid shell, the intensity as well as the sharpness of the sphere outline is much higher in T2-weighted images as depicted in Figure 1. However, the positioning as well as the spacing error do not suffer from differences in contrast or image intensity due to different imaging modalities, as can be seen in Figure 8 and Figure 9. When considering the positioning error, a slightly higher overall maximum median error of $E_{p,max}=2.37\;\mathrm{mm}$ (kernel method at s=1.4mm) can be observed for T1-weighted images compared to the results from T2-weighted image, which may be addressed by the previously mentioned reasons.

Besides the spatial errors, the classification accuracy doesn’t vary significantly between the two chosen modalities. Only the Hough-method shows a higher $F_{1}$ score before application of the marker model for the T2 images for all voxel sizes, due to a lower number of falsely positive detected spheres, especially for larger voxel sizes from 1.0mm to 1.6mm. Since this method is explicitly sensitive to circular shapes in the 2D slices of the volume, a well defined and sharp outline of the spheres is of particular importance and more present in the T2-images. For the blob method, T2 images seemingly produce a much higher number in falsely positive sphere candidates for some voxel sizes, which is reflected in relatively low $F_{1}$ score. Accordingly, the lowest score for all methods of ${F_{1}}_{\mathrm{bm}}=0.19$ is met by the blob-method for a T2 image at a voxel size of 0.6mm. The high number of false positives could be caused by the 2D manner of image processing in case of the blob-method. By slicing the volumetric image in a specific direction, structures in the brain or bone tissue may appear circular at an specific plane due to its relative orientation, even though the 3D geometry of the concerned structure is not spherical but e.g., cylindrical or tube-like. This holds also true for the Hough-method which also works on 2D image data. However, this method produces fewer false positive sphere candidates because the preliminary result from the first slice direction is amplified or attenuated by the remaining slice directions, which discards a higher number of false positive sphere candidates.

### 4.2. Influence of the Voxel Size

The voxel size has a major impact on the spatial error of the detected markers. This influence is mainly driven by the ration between the size of the voxel and the physical size of the spheres. The higher the voxel size, the less voxels contribute to a marker sphere. Since the algorithms calculate the spheres centroid on a discrete grid, whose resolution is determined by the voxel size, this may result in a bigger spatial error, if the real position of the spheres centroid does not match the center of a voxel. Thus, the resolution limit can be approximated as half a voxel in every translatory degree of freedom which results in an approximate limiting of d=0.86·s, where *s* is the voxel size. For voxel sizes of s≤1.0mm all methods except the Kernel-method are able to detect the markers position within a median positioning error which is smaller then the proposed theoretical limit. For voxel sizes of s>1.0mm, the positioning error increases, which is especially the case for the blob- and kernel-method, having a maximum median error of $E_{p}\;=\;2.21\;\mathrm{mm}$ and $E_{p}\;=\;2.37\;\mathrm{mm}$, respectively. The spacing error remains relatively constant over the full range of chosen voxel sizes and never exceeds a maximum median value of $E_{s}\;=\;1.48\;\mathrm{mm}$ in case of the blob-method. Since the spacing error was only calculated for complete markers, i.e., two spheres which fulfill a maximum spacing criterion stated in the marker model, the spacing error is accordingly lower then the positioning error. Besides the accuracy of the methods, the voxel size influences the computational effort as well. Smaller voxel sizes lead to bigger image volumes and therefore a higher amount of data which needs to be processed. However, the blob detection method is slower by two orders of magnitude compared to the other methods, over the whole range of chosen voxel sizes. This may be caused by the high number of possible sphere candidates produced by the DoH and the resulting computational effort in the subsequent filtering steps.

### 4.3. Influence of the Marker Model

The marker model basically serves the function to discard falsely positive spheres, detected by the algorithms, by classifying them as marker sphere. On the one hand, a marker must consist of two spheres, i.e., to every detected sphere there must be a single second matching sphere in the local neighborhood. On the other hand, the spacing between those spheres must satisfy a well defined threshold corridor. Accordingly, the $F_{1}$ score was calculated before and after application of the marker model to emphasize the influence of this classification step. Thus, the application of the marker model increases the $F_{1}$ score in cases where the method detects a large number of false positive spheres. Since the Hough- and the blob-method tend to detect more false positive spheres then the other methods, both results benefit from the application of the marker model, with a maximum $F_{1}$ score increase factor of $\frac{{F_{1}}_{\mathrm{am}}}{{F_{1}}_{\mathrm{bm}}}=2.1$.

However, the classification step can also lead to a decrease of the $F_{1}$ score by discarding actually true positive spheres, which do not satisfy the marker model constraint in the given threshold corridor. The spatial threshold in the marker model does not vary with the voxel size. In contrast the centroid position of the detected spheres is determined to the center of a voxel by the algorithms. Due to this fact, the variation between two neighboring voxels can lead to large spatial errors in terms of physical coordinates, especially for large voxel sizes.

### 4.4. Error of Orientation Estimation

The orientation of the marker main axis is defined by the centroids of the two corresponding marker spheres and thus suffers from the positioning errors occurring in any spatial direction. In order to estimate the orientation error, we assumed a positioning error orthogonal to the markers actual main axis. This assumption leads to the worst effect on the estimated orientations. By distinguishing between data sets acquired at voxel sizes lower or equal 1 mm and voxel sizes above, we valued the effect of the increasing positioning error related to an increasing voxel size. This assumption leads to mean angular errors for voxel sizes ≤1 mm (>1 mm) of 1.5 (3.2) for CCA, 5.5 (15.7) for blob, 4.7 (7.8) for Hough and 8.9 (13.4) for kernel method, each given in degrees.

## 5. Conclusions

By developing and comparing four different methods for spherical fiducial detection in both, T1- and T2-weighted images acquired at different voxel sizes, we have found that the Connected Component Analysis serves the most robust and accurate results. The method is capable of reliably detecting spheres with a mean $F_{1}$ score of f1=0.97, averaged over all measurements. Localizing the spheres centroids was accomplished highly accurate with a mean error of 0.22mm which is better then reported localization errors in the literature of 0.40mm [7] on clinical and 0.31mm [34] on non-clinical data. Regarding the smallest voxel size of 0.6mm chosen, we have been able to detect the sphere centroids in clinical data within sub-voxel accuracy.

In contrast to previously proposed marker designs, we have chosen a spherical shape of the fiducials instead of cylindrical [7] or toroidal [35], which allows reliable marker detection in arbitrary orientation. The experiments have shown, that the imaging modality does not have a major impact on the detection result. This circumstance allows the promising potential use of the methods for spherical fiducial detection in other imaging techniques such as microscopy or CT.

Even though our proposed approaches, among previously proposed methods [1,7], must be considered knowledge based. The only user set parameters are the diameter of the spheres for single sphere detection and the spacing in case of application of the marker model. Thus, the methods and marker design are easy to adapt and applicable to other use-cases.

Future work, should therefore take into account use-case specific characteristics, e.g., how a probably altered signal-to-noise ratio may influence the capabilities of the proposed methods. In the manifold of governing image-acquisition parameters, we identified the underlying voxel size as most result influencing image parameter and discussed its impact on position and classification accuracy and precision. Accordingly, we have found the voxel size being the biggest limiting factor in terms of spatial detection accuracy. Since the detection of the sphere center works in a voxel-based manner, voxel sizes above 1.0mm result in a positioning error increase.

Our method, along with the proposed marker model, allows the point-based registration of T1- and T2-weighted images as well as the detection of cranial bone anchors without any further CT-imaging and thus is a promising technique for e.g., stereotactic surgery navigation.

## Figures and Tables

**Table 1:** Classification results, sorted by image modality (mod), voxel size (s) and method (Connected Component Analysis (CCA), kernel-based (kernel), circular Hough transform (Hough), blob detection related (blob)). Detected spheres where classified as true positive (tp) if the Euclidean distance between the detected centroid and the ground-truth measurement satisfies a threshold of 14mm, otherwise they were classified as false positive (fp). Spheres present in the ground truth measurement but not detected by the algorithms were classified as false negative (fn). This classification task was conducted before applying the marker model. The according $F_{1}$ score was calculated before (${F_{1}}_{\mathrm{bm}}$) and after (${F_{1}}_{\mathrm{am}}$) application of the marker model (the marker must contain two spheres at a certain distance). $F_{1}$ scores close to 1 are better, see Equation (5).

Mod	s [mm]	CCA					Kernel					Hough					Blob				
		tp	fp	fn	${F_{1}}_{\mathrm{bm}}$	${F_{1}}_{\mathrm{am}}$	tp	fp	fn	${F_{1}}_{\mathrm{bm}}$	${F_{1}}_{\mathrm{am}}$	tp	fp	fn	${F_{1}}_{\mathrm{bm}}$	${F_{1}}_{\mathrm{am}}$	tp	fp	fn	${F_{1}}_{\mathrm{bm}}$	${F_{1}}_{\mathrm{am}}$
T1	0.6	10	0	0	1.00	1.00	10	1	0	0.95	1.00	10	9	0	0.69	1.00	10	6	0	0.77	1.00
T1	0.8	10	0	0	1.00	1.00	10	0	0	1.00	1.00	10	3	0	0.87	1.00	10	38	0	0.34	0.53
T1	1.0	10	0	0	1.00	1.00	10	0	0	1.00	1.00	10	3	0	0.87	0.89	10	0	0	1.00	1.00
T1	1.2	9	0	1	0.95	0.89	10	0	0	1.00	0.75	10	20	0	0.50	0.80	10	1	0	0.95	1.00
T1	1.4	9	0	1	0.95	0.89	3	1	7	0.43	0.00	10	16	0	0.56	0.89	10	1	0	0.95	1.00
T1	1.6	9	3	1	0.82	0.89	5	0	5	0.67	0.33	10	20	0	0.50	0.89	10	0	0	1.00	1.00
T2	0.6	10	1	0	0.95	1.00	10	0	0	1.00	1.00	10	2	0	0.91	0.91	10	87	0	0.19	0.40
T2	0.8	10	0	0	1.00	1.00	10	1	0	0.95	1.00	10	3	0	0.87	0.91	10	60	0	0.25	0.53
T2	1.0	10	0	0	1.00	1.00	10	0	0	1.00	1.00	10	1	0	0.95	1.00	10	0	0	1.00	1.00
T2	1.2	10	1	0	0.95	1.00	7	0	3	0.82	0.75	10	1	0	0.95	0.57	10	0	0	1.00	0.89
T2	1.4	10	1	0	0.95	1.00	7	0	3	0.82	0.75	10	2	0	0.91	0.89	10	76	0	0.21	0.43
T2	1.6	10	3	0	0.87	0.91	5	0	5	0.67	0.33	10	6	0	0.77	1.00	10	2	0	0.91	0.89

## Data Availability

The data presented in this study are available on request from the corresponding author. The data are not publicly available due to privacy restrictions.
